# La utilidad y el impacto de incorporar la investigación sobre implementación para mejorar la ejecución de los programas: perspectivas de América Latina y el Caribe^*^

**DOI:** 10.26633/RPSP.2021.117

**Published:** 2021-08-31

**Authors:** Robert Marten, Ludovic Reveiz, Garry Aslanyan, Freddy Perez, Abdul Ghaffar

**Affiliations:** 1 Alianza para la Investigación en Políticas y Sistemas de Salud, Organización Mundial de la Salud Ginebra Suiza Alianza para la Investigación en Políticas y Sistemas de Salud, Organización Mundial de la Salud, Ginebra, Suiza; 2 Organización Panamericana de la Salud Washington, DC Estados Unidos de América Organización Panamericana de la Salud, Washington, DC, Estados Unidos de América; 3 Programa Especial de Investigación y Capacitación en Enfermedades Tropicales, Organización Mundial de la Salud Ginebra Suiza Programa Especial de Investigación y Capacitación en Enfermedades Tropicales, Organización Mundial de la Salud, Ginebra, Suiza

La incorporación de la investigación se realiza como una parte integrada y sistemática de la toma de decisiones y la formulación y ejecución de políticas en materia de salud (1). Implica una colaboración continua entre los encargados de las decisiones, los investigadores y las comunidades. La incorporación de la investigación se centra en los cambios a nivel de los sistemas y arroja luz sobre los factores específicos de cada contexto que influyen en los programas, las políticas y las decisiones sobre sistemas relacionados con la salud en el mundo real. En consecuencia, mejora el sentido de apropiación y la ejecución (2). Con esto en mente y en el marco de una iniciativa conjunta, la Alianza para la Investigación en Políticas y Sistemas de Salud, la Organización Panamericana de la Salud (OPS) y el Programa Especial de Investigación y Capacitación en Enfermedades Tropicales (TDR) de la Organización Mundial de la Salud (OMS) hicieron una convocatoria para que los investigadores y los responsables de las políticas y las decisiones indicaran las razones por las cuales es necesario incorporar las investigaciones para mejorar la ejecución de programas a fin de acelerar el progreso hacia los Objetivos de Desarrollo Sostenible (ODS) en América Latina y el Caribe, y proporcionaron cooperación técnica a lo largo de todo el proceso. Tras la convocatoria para presentar propuestas y el examen de las más de 200 propuestas de investigación recibidas, se seleccionaron 13 equipos de 11 países de ingresos bajos y medianos de América Latina y el Caribe (Argentina, Bolivia, Brasil, Colombia, Ecuador, Guatemala, Guyana, Haití, Paraguay, Perú y la República Dominicana). En este suplemento de la *Revista Panamericana de Salud Pública* se presentan las conclusiones de estos estudios sobre la incorporación de la investigación en la ejecución de los programas. Se pone de relieve la utilidad de la incorporación de la investigación y se detalla el impacto de este tipo de trabajo, con ejemplos concretos. Los resultados de los artículos que se presentan en este suplemento indican una necesidad cada vez más reconocida de desarrollar la capacidad para colaborar por medio de la acción multisectorial con el fin de alcanzar los ODS.

Sobre la base de las innovaciones metodológicas de la estrategia de la OMS para la investigación en políticas y sistemas de salud (2012) y el *Informe sobre la salud en el mundo* de la OMS del 2013, en el cual se abordó la investigación orientada a la cobertura universal de salud, y de la colaboración previa entre la OPS, la Alianza y el Programa Especial de Investigación y Capacitación en Enfermedades Tropicales, la incorporación de la investigación ha pasado a ser un enfoque que los responsables de la formulación de políticas cada vez valoran más para conceptualizar y ejecutar intervenciones de salud. La actual pandemia de COVID-19 es un ejemplo de los estrechos vínculos entre las políticas y la investigación (3), así como de la necesidad de una interdisciplinariedad mucho mayor (4). Al resumir su labor como centro de apoyo técnico, Becerril-Montekio et al. afirman que esa experiencia y los resultados que se presentan en este número especial confirman el valor de este tipo de iniciativas. Mariani et al. evalúan las funciones de la atención primaria de salud desde la perspectiva de los pacientes con tuberculosis de barrios marginales de la ciudad de Buenos Aires (Argentina) y proporcionan ideas sobre la forma de mejorar estos servicios (5). En otro ejemplo, Polanco-Pasaje et al. evalúan la atención de la tuberculosis en la población indígena de Colombia y señalan las lagunas (6).

Estos estudios de investigación también documentan la manera en que la incorporación de la investigación puede influir en la ejecución de las políticas y mejorarla. Por ejemplo, Diez-Canseco et al. analizan el establecimiento de centros de salud mental en Perú y destacan su impacto en las comunidades locales (7). Barreto et al. detallan la forma en que la investigación mejoró la elaboración de las directrices nacionales sobre el parto en Brasil y, específicamente, cómo la investigación ayudó a detectar y superar obstáculos para la ejecución (8), además de explicar en detalle que la capacitación y la cultura profesionales, la cultura social y las cuestiones políticas y de gestión son aspectos cruciales que deben tenerse en cuenta (9).

Los estudios sobre la incorporación de la investigación que se presentan en este suplemento también muestran la importancia fundamental de fomentar la participación de las comunidades y cultivar la capacidad de colaborar con otros sectores a fin de alcanzar los objetivos buscados en el ámbito de la salud. Laureano-Eugenio et al., al examinar la estrategia de Municipios Saludables de Guatemala para mejorar su ejecución, documentaron el potencial de mejorar en la promoción y el fortalecimiento de la participación social, así como la insuficiencia de las inversiones en la labor relacionada con los determinantes sociales de la salud (10). En otro ejemplo, Sacoto et al. estudiaron una iniciativa para prevenir enfermedades no transmisibles en Ecuador y observaron que una institucionalización insuficiente podría limitar la sostenibilidad del esfuerzo y que era necesario mejorar la coordinación de la iniciativa con otros sectores y actores (11). Para codificar y mejorar la forma en que esto se hace, como otros han señalado, sigue siendo necesario apoyar el intercambio de experiencias en la acción multisectorial en otros ámbitos fuera de la salud (12).

Si bien todavía hay margen para intensificar la acción a fin de cerrar la brecha entre los investigadores y los responsables de las políticas y las decisiones, los artículos de esta serie demuestran el valor y el impacto de la incorporación de la investigación para mejorar la ejecución programática. Este suplemento también pone de relieve los desafíos persistentes, como el trabajo con las comunidades y la necesidad de tener en cuenta la multisectorialidad a fin de avanzar en las metas en materia de salud. A fin de avanzar en este tipo de trabajo, se requieren nuevos enfoques para apoyar la investigación. Es necesario pasar de proyectos de investigación *ad hoc* a un mayor énfasis en la formación de alianzas más colaborativas y a más largo plazo para posibilitar la incorporación de la investigación. La Alianza para la Investigación en Políticas y Sistemas de Salud, el Programa Especial de Investigación y Capacitación en Enfermedades Tropicales de la Organización Mundial de la Salud y la Organización Panamericana de la Salud están comprometidos a avanzar en esta dirección.
